# Discovery and Characterization of Uracil Derivatives Targeting the Set-and-Ring Domain of UHRF1

**DOI:** 10.1021/acs.jcim.5c01345

**Published:** 2025-08-19

**Authors:** Ifigeneia Akrani, Danai Driva, Efstratios Tsakalidis, Nandini Mozumdar, Danai Mavridi, Anthi Panara, Kalypso Epiphaniou, Maria Chalkiadaki, Andreanna Frances Wright, Maria Halabalaki, Angeliki Tsoka, Grigoris Zoidis, Constantinos Vorgias, Duncan Sproul, Evangelos Gikas, Skirmantas Kriaucionis, Emmanuel Mikros, Vassilios Myrianthopoulos

**Affiliations:** † Division of Pharmaceutical Chemistry, Department of Pharmacy, 68993 National and Kapodistrian University of Athens, Panepistimiopolis Zografou 15771, Athens, Greece; ‡ Ludwig Institute for Cancer Research, Nuffield Department of Medicine, Old Road Campus Research Building, OX3 7DQ, Oxford, United Kingdom; § Laboratory of Analytical Chemistry, Department of Chemistry, 68993 National and Kapodistrian University of Athens, Panepistimiopolis Zografou 15771, Athens, Greece; ∥ Medical Research Council Human Genetics Unit, Institute of Genetics and Cancer, 59892 University of Edinburgh, EH4 2XU, Edinburgh, United Kingdom; ⊥ Department of Biology, 68993 National and Kapodistrian University of Athens, Panepistimiopolis Zografou 15771, Athens, Greece

## Abstract

The Set and Ring domain of the UHRF1 oncogene is responsible for its interaction with hemimethylated DNA and faithful propagation of epigenetic signaling over cellular replication. Inhibiting this recognition can have serious implications for UHRF1 functionality and may possibly enable therapeutic interventions. Based on a previous finding indicating a promising *in vitro* DNA demethylating potential of a pyrimidine derivative, a subscaffold search was performed in the NCI/DTP compound repository to discover similar molecules and evaluate their affinity for the SRA domain of UHRF1. Toward this direction, several compounds were evaluated using a thermal melt screen, and the most promising hits were subsequently studied by calorimetry in terms of their capacity to bind the 5-methylcytosine recognition site of UHRF1. A markedly different thermodynamic profile between the two confirmed hits with an intense enthalpy–entropy compensation signature was determined. The systems were further studied by biased and unbiased molecular simulations, computational hydration mapping, and calorimetry-based heat capacity measurements to devise a hypothesis on the structural requisites for efficient SRA binding. The most potent compound was evaluated for its DNA methylation effects against the UHRF1-dependent colorectal cancer HCT116 cells, where promising global demethylating activity reaching an approximate 75% reduction compared to control was achieved after treatment with 25 μM of NSC232005. Based on the presented results, rationally substituted analogues of the uracil scaffold appear as highly promising UHRF1 modulators for exploring its diverse functionalities and validating the protein as a drug target.

## Graphical abstract

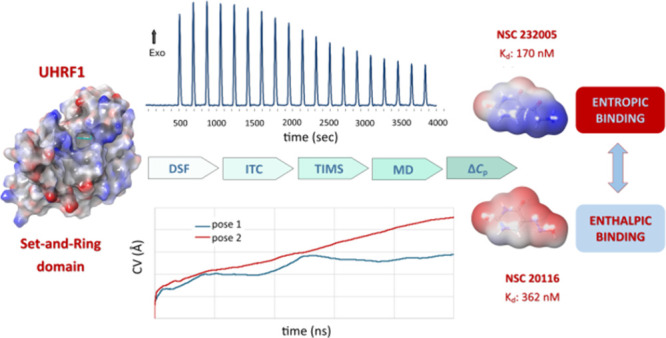

## Introduction

Epigenetics is currently recognized as a particularly exciting scientific field at the interface between core biology and therapeutics. The increasing medical interest in epigenetic mechanisms and their implications in human health arise from collective experimental evidence indicating their multisided involvement in fundamental biological processes such as cellular growth, differentiation, immune responses, and malignant transformation.^1–4^ Medical interventions directly targeting malfunctioning epigenetic modules are highly promising novel approaches for treating serious pathologies such as cancer and neurological and inflammatory diseases.^5–10^ Among the most well-studied targets are enzyme families involved in writing or erasing the epigenetic code on DNA and histones. Histone methyltransferases such as EZH2, demethylases like LSD1, numerous members of the JMJ family including KDM3, the histone acetyltransferases (HATs), the histone deacetylases HDACs and SIRTs, as well as the DNA methyltransferases (DNMTs) are now validated drug targets.^11–21^ Moreover, families of epigenetic code reader modules, such as the bromodomains (BRDs), chromodomains, and YEATS, are continuously pursued as promising and highly druggable protein–protein interaction targets (PPI), while several BRD inhibitors, especially of the PROTAC family, such as FHD-609 and CFT8634, are evaluated in clinical trials against several indications.^22^

Nowadays, cumulative data support the hypothesis that the DNA methylation maintenance factor Ubiquitin-like containing PHD and RING Finger domains 1 (UHRF1) is a protein of pivotal importance in the integration of epigenetic signals from both DNA methylation and histone post-translational modifications. Its main functionality is to facilitate faithful inheritance of the epigenetic code through maintaining the methylation status of newly synthesized DNA.^23^ Crucial to this series of events is recognition of hemimethylated DNA by UHRF1 and subsequent recruitment and localization of DNA methyltransferase machinery (DNMT1) onto the newly synthesized DNA via ubiquitination.^24–26^ Furthermore, a number of studies have shown that UHRF1 is involved in a diverse array of physiological processes. Its knockdown leads to cell cycle arrest, enhanced apoptosis and activation of the DNA damage response (DDR) pathway, leading to cell cycle arrest in G2/M phase and caspase-dependent apoptosis.^27,28^ UHRF1 interacts with a wide array of partners.^29^ This multifaceted capacity for interactions is justified by the fact that UHRF1 is a multidomain protein comprising a number of distinct domains including an N-terminal ubiquitin-like domain (UBL), a Tandem Tudor Domain (TTD) and a Plant Homeodomain (PHD) both involved in binding of methylated histones (H3K9me2/3 and H3R2, respectively), a Set and Ring (SRA) domain that is responsible for 5-methylcytosine (5mC) recognition and subsequent hemimethylated DNA (hmDNA) binding, and a C-terminal Really Interesting New Gene (RING) domain with E3 ligase activity. The SRA is the first studied domain and crystal structures of SRA-UHRF1 complexed with DNA, unmodified and methylated histone H3 tail peptides have been determined.^25,30^ The PHD and TTD domains have also been studied by X-ray crystallography and NMR.^31–33^ In terms of structure, it was recently shown that UHRF1 exists in a dynamic equilibrium between an open and a closed conformation.^34^ Binding of UHRF1 to hemimethylated DNA (hmDNA) gives rise to an equilibrium shift toward the open conformation, which can subsequently function as an active mediator of epigenetic signaling. This very interesting aspect of UHRF1 internal dynamics might have a serious influence on inhibitor development endeavors.

The UHRF1 gene is frequently overexpressed in a number of cancers and its deregulation has been widely correlated with a variety of disease phenotypes.^29,35,36^ Growing evidence link its aberrant functionality with malignancy development, hence leading to its establishment as an oncogene and a potential biomarker.^37,38^ Indeed, one of the most interesting aspects of UHRF1 involvement in malignant transformations is methylation and silencing of tumor suppressor genes such as *BRCA1*, *p73*, *PPARγ* and, most importantly, *p16* ^*INK4A*^.^39–43^ UHRF1 is currently identified as a key driving factor of several malignancies, such as melanoma, breast, bladder, colon, cervical, pancreatic and prostate cancer, leukemia, and lung or hepatocellular carcinoma.^37,44–52^ As such, UHRF1 comprises a highly promising target for therapeutic intervention.^53^ The various physiological functions of UHRF1 demonstrate a clear cell-cycle dependency that is regulated by ubiquitination. Interaction between UHRF1 and chromatin is facilitated by binding of USP7, a deubiquitinase.^54^ Furthermore, UHRF1 has been identified as a client protein for HSP90 chaperone and it was suggested that inhibitors of HSP90 may suppress cancer cell proliferation partly by inducing UHRF1 degradation.^55^ The interaction between UHRF1 and DNMT3A/B^56,57^ as well as various less explored functions such as UHRF1 regulation of retrotransposon silencing^58^ or crosstalk between UHRF1 and the histone lysine *N*-methyltransferases SUV39H1/H2 which affect tumor suppressor genes in colorectal cancer^59^ are among newly discovered UHRF1 features, further strengthening its putative significance as a tractable therapeutic target.

Although the therapeutic potential of small-molecule UHRF1 modulators is strongly suggested by a consensus of evidence both at the cellular and whole organism levels, a very limited number of studies have reported the discovery of small molecule UHRF1 inhibitors. Indirect ways for targeting UHRF1 have been proposed, such as HSP90 inhibition which leads to ubiquitination and proteasome-dependent UHRF1 degradation.^55^ Yet, direct interaction of small molecules with the various UHRF1 domains remains a top priority for targeting UHRF1 in a therapeutic fashion due to their high apparent druggability, especially of the TTD, PHD and SRA. Indeed, a limited number of compounds have been described as PHD domain inhibitors, while research is ongoing on TTD domain inhibitors with compounds F1957–0088 (IC_50_: 45.1 ± 5.4 μM), 2,4-lutidine (IC_50_: 29.2 ± 1.4 μM), NV01 (*K*_d_: 5.2 ± 1.2 μM, stoichiometry 1:1), and NV03 (*K*_d_: 2.4 ± 0.2 μM, stoichiometry 1:1) reported as TTD binders.^60,61^ Regarding SRA, a very limited array of compounds have been shown to directly bind the domain, such as the anthraquinone UM63 (*K*_d_ of 0.94 ± 0.25 μM, stoichiometry of 1:1) and follow-up derivatives AMSA2 (a hydroxyanthracene with IC_50_ of 5.4 ± 0.2 μM) and MPB7 (an imidazoquinoline with IC_50_ of 20.0 ± 1.0 μM).^62,63^ Indirect assays have identified clinically used anticancer drugs doxorubicin (IC_50_: 0.131 μM), idarubicin (IC_50_: 0.593 μM), daunorubicin (IC_50_: 0.313 μM), mitoxantrone (IC_50_: 0.134 μM), and pixantrone (IC_50_: 0.557 μM) as UHRF1-hmDNA disruptors, yet without evidence of direct binding to the 5mC cavity of SRA-UHRF1.^64^

Our group has reported the discovery of compounds possessing DNA-demethylating activity by targeting the aforementioned domain of UHRF1, with the most potent analog being a pyrimidine derivative.^65^ To our knowledge, these were the first compounds reported to target SRA-UHRF1, thus providing primary yet strong evidence of the domain druggability. Given the pivotal position of UHRF1 in the mechanisms of epigenetic regulation, in this study we further pursue this particular heterocyclic scaffold to show that specific uracil derivatives are potent ligands of the SRA domain of UHRF1, we investigate the structural and thermodynamic requisites of their binding affinity and we provide preliminary proof-of-concept for their global DNA demethylation capacity in an *in vitro* setting.

## Results and Discussion

### Initial Screening and Isothermal Titration Calorimetry Measurements

Aiming to a more systematic exploration of our previously reported uracil- and indole-based SRA-UHRF1 ligands^65^ in terms of structure–activity relationships (SAR), a subscaffold search was performed within the DTP/NCI Repository to discover derivatives with structural resemblance to the above-mentioned compounds. The web module of PubChem for molecular similarity assessment was utilized and selection of compounds was performed on the basis of highest Tanimoto score, visual inspection and sample availability at the NCI Repository.^66^ A set of 20 top-ranked closely related compounds were obtained (Table S1) and evaluated as potential SRA-UHRF1 binders by differential scanning fluorimetry (DSF). The set was augmented with the monomer of the native ligand, 5-methyl-2’-deoxycytidine-5′-triphosphate (5-Me-dCTP). Most of the screened molecules afforded weak thermal shifts with both stabilizing and destabilizing effects on the SRA domain, as interpreted by their Δ*Τ*_m_ values (Figure 1A). However, a routine search within the screened group for structures with poor development potential and pan-assay interference features (PAINS)^67,68^ resulted in several instances of suboptimal molecular scaffolds which were hence eliminated from further experimental validation. More specifically, analogues demonstrating otherwise statistically significant Δ*T*_m_ values carried undesirable lead-like features such as poor water solubility (NSC107682), PAINS structural characteristics related to toxicity concerns (NSC107684 - thiocarbonyl derivative)^67^ as well as obvious assay interference issues (NSC22474 and NSC22475, yellow-colored molecules interfering with orange fluorescent DSF dye; NSC232002, high intrinsic fluorescence - please see Materials and Methods). The two uracil analogues affording the most intense negative *Τ*_m_ shift with no PAINS concerns, molecules NSC232005 and NSC20116 (Figure 1B), were advanced to a calorimetric confirmation of their binding affinity toward SRA-UHRF1. Although Δ*T*_m_ values tend to be positive in protein–ligand interactions, negative shifts have been reported in several proteins for confirmed binding events.^69–72^ In those instances, however, an orthogonal confirmation is needed to verify this interaction. In our system, the isothermal titration calorimetry experiments confirmed both hits as UHRF1-SRA ligands, with NSC232005 affording a *K*_d_ value of 170 ± 89 nM (at 25 °C) and a 1:1 stoichiometry (*n*: 1.32 ± 0.04), whereas NSC20116 afforded a *K*_d_ value of 362 ± 181 nM (at 25 °C) and a 1:1 stoichiometry (*n*: 1.13 ± 0.05) based on fitting data to an independent binding model (Figure 1B). In terms of thermodynamics, though, a markedly different profile was determined between the two compounds regardless of their structural similarity (Figure 3B). More specifically, binding of NSC232005 to SRA-UHRF1 demonstrated a rather weak thermal signature (Δ*H*: −2.60 ± 0.11 kcal/mol) along with a slightly more dominant yet constructive entropic term (−*T*Δ*S*: −6.63 kcal/mol). Conversely, NSC20116 afforded high binding enthalpy (Δ*H*: −15.96 ± 0.94 kcal/mol) along with an unfavorable entropy (−*T*Δ*S*: 7.17 kcal/mol). Notably, the binding affinity of both compounds resulted in relatively high ligand efficiency (LE) values, with 0.77 and 0.80 kcal/mol of binding free energy change attributed to each heavy atom for NSC232005 and NSC20116, respectively. The compounds were subjected to a dose–response analysis, where a consistent series of shifts was obtained over concentrations between 10 and 1000 μM (Figure 1D). Notably, the lack of a perfect sigmoidal response has been described several times for similar dose–response results produced by DSF.^71^ Of interest, among the screened compounds, the uracil metabolite orotic acid (NSC9791) erroneously thought in the past to be a B-complex vitamin (B13), resulted in a negative *T*_m_ shift of −1.36 ± 0.21 °C, although this hit was not pursued beyond the initial screening.

**Figure 1:**
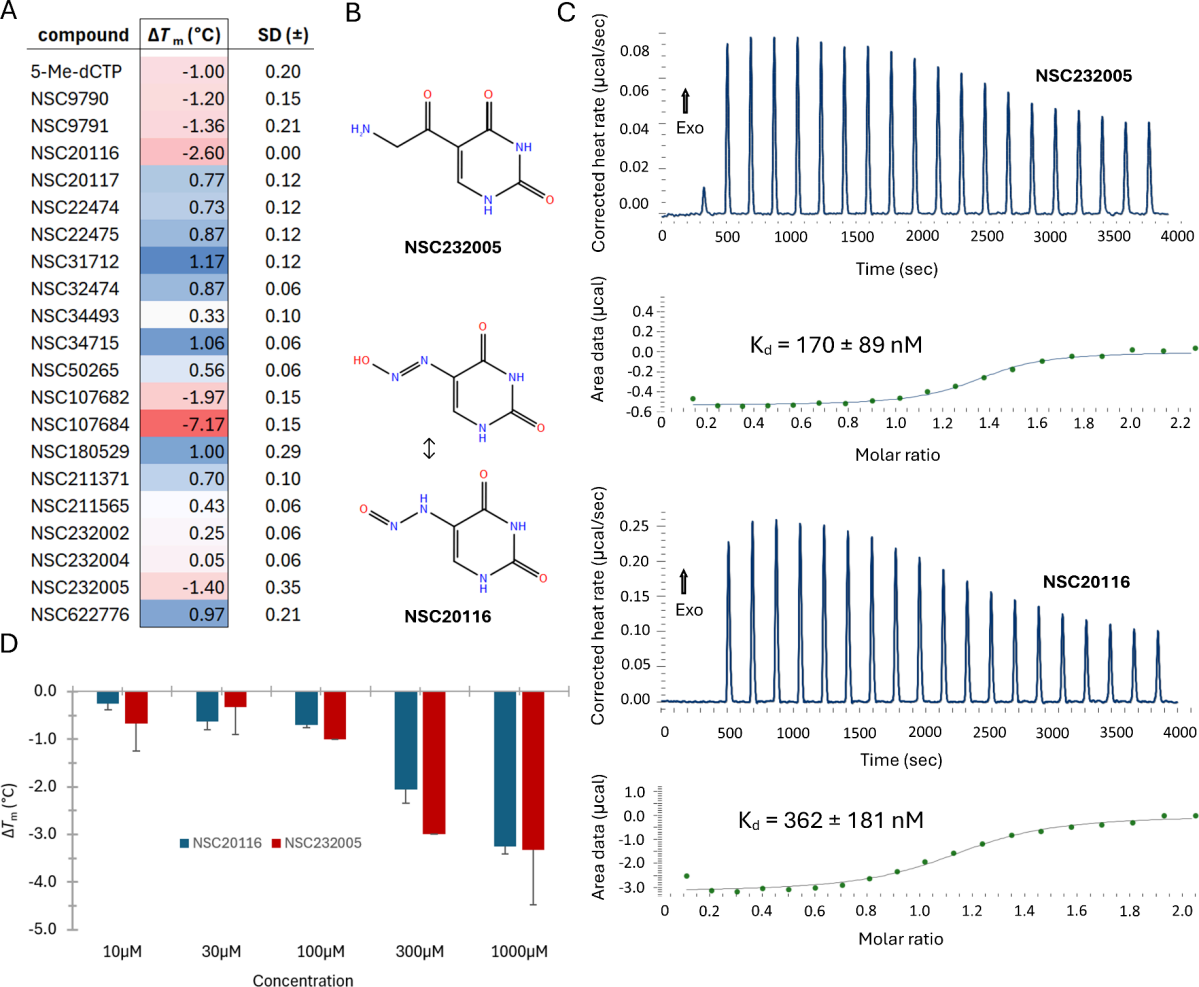
A summary of the primary DSF screening and subsequent ITC confirmation of uracil hits. (A) Heat map of the screened panel of compounds with the respective names and NCI/DTP numbers as well as the related *Τ*_m_ shifts with their standard deviations (*n* = 3). (B) Two-dimensional structures of the two confirmed hits, compound NSC232005 and the two dominant tautomeric forms of NSC20116. (C) The ITC thermograms and fitted models of the calorimetric study for each of the confirmed primary hits. (D) The effect of NSC20116 and NSC232005 at different concentrations on the *T*_m_ shift of the SRA-UHRF1 domain (*n* = 3).

### Molecular Simulations

The interesting ITC data prompted for a more systematic investigation of binding requisites concerning these hits, as each one appears to bind with opposing thermodynamic features; enthalpic contributions in NSC20116 versus entropic gain in NSC232005 seemingly determine the binding affinity of each complex. To provide a structural rationale for the apparent enthalpy–entropy compensation events related to these ligands, molecular simulations were undertaken. Compound NSC232005 is a uracil derivative bearing an exocyclic primary aliphatic amine. The primary amine of NSC232005 is expected to be protonated and unaffected by the possible tautomer equilibrium of the core pyrimidine system. Utilization of *ab initio* calculations and a (de)­protonation/(de)­solvation thermodynamic cycle^73–75^ confirmed this notion by indicating a basic p*K*_a_ value of 8.53 for the amine N atom. The exact tautomeric state of NSC232005 was also explored by high-level quantum-mechanical calculations at the DFT level of theory. Simulations unambiguously indicated the diketo as the most stable tautomer, by 12.8 and 5.1 kcal/mol from the second most stable di-enol isomer in the gas and solution phase, respectively. Yet, concerning NSC20116 two energetically favorable tautomers were present. The hydroxydiazenyl state was more stable in solution by 1.6 kcal/mol compared to its nitroso isomer, although the opposite ranking was determined in the gas phase with a difference of 2.3 kcal/mol. Evidence for the actual presence of two major tautomers in NSC20116 was obtained experimentally by Trapped Ion Mobility Mass Spectrometry (IM-MS), demonstrating two major species with different motion characteristics against the spectrometer gas current, thus providing additional confirmation to the DFT simulations (Figure 2E). The possibility of intramolecular hydrogen bonding was also assessed for both compounds, but this scenario was ruled out on the basis of DFT energies calculated for the respective species with and without internal bonding (Figure S1). The dominant cationic tautomer of NSC232005 and both NSC20116 neutral tautomers (designated as 20116-H and 20116-N, respectively, and treated thereafter as different entities in all simulations) were hence docked to the 5mC pocket of SRA-UHRF1 (Glide XP software, Schrodinger Inc.) and 9 low energy binding poses were obtained (Figure 2A).^76–78^ Metadynamics were used as the next step for assessing the stability for each of these geometries. The collective variable (CV) in these simulations was the root-mean-square deviation from starting coordinates of the ligand over 10 short MD trajectories of 10 ns each according to a previously reported procedure (Figure 3B).^79^ The most stable pose of each ligand in complex with SRA-UHRF1 as determined by metadynamics was subsequently studied in terms of stability, intermolecular interactions and conformational dynamics over three independent trajectories of 1 μs each, using unrestrained Molecular Dynamics and Desmond software (D.E. Shaw Research Inc.).^80^ Solvent mapping calculations utilizing the SZmap algorithm (Openeye Inc.) were also used in combination with the MD simulations to suggest an explanation as to the recorded enthalpy–entropy compensation regarding the two structurally related pyrimidine hits.^81^

**Figure 2:**
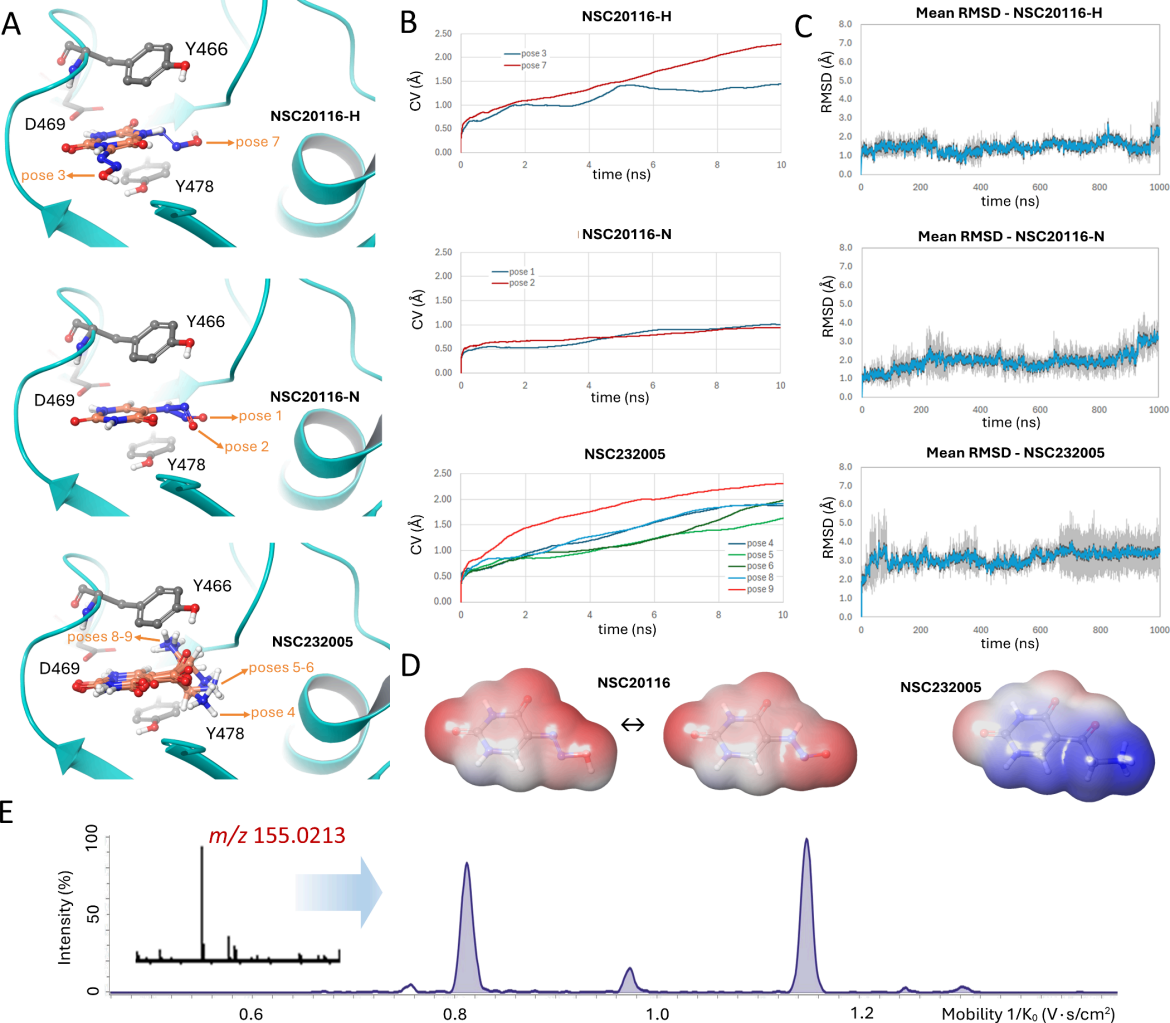
Calculations at different levels of theory and MS experiments were utilized to provide a rationale for the observed enthalpy–entropy compensation effects determined for the binding of the two hits. (A) The most stable binding poses of each ligand and tautomer, as determined by rigid docking. (B) The metadynamics evaluation of each docked pose stability as a function of the ligand deviation from starting coordinates (CV) over 10 simulations of 10 ns each. (C) The stability of each ligand in the 5 mC binding cavity, as determined by the root-mean-square deviation over three independent unbiased MD simulations of 1 μs each, with the average depicted as a blue line and the standard deviation as a gray interval (*n* = 3). (D) The electrostatic potential mapped on the 0.001 au electron density isosurfaces of the cationic NSC232005 and the two dominant neutral tautomers of NSC20116 (common ESP scale applies). (E) The TIMS (trapped ion mobility spectrometry) - MS mobilogram of NSC20116 (*m*/*z* of base peak [M – H]^−^ 155.0213 demonstrated as inset), indicating two major species and their respective populations possessing varied drift values (expressed as 1/*K*_0_) through the trapped ion mobility buffer gas.

**Figure 3:**
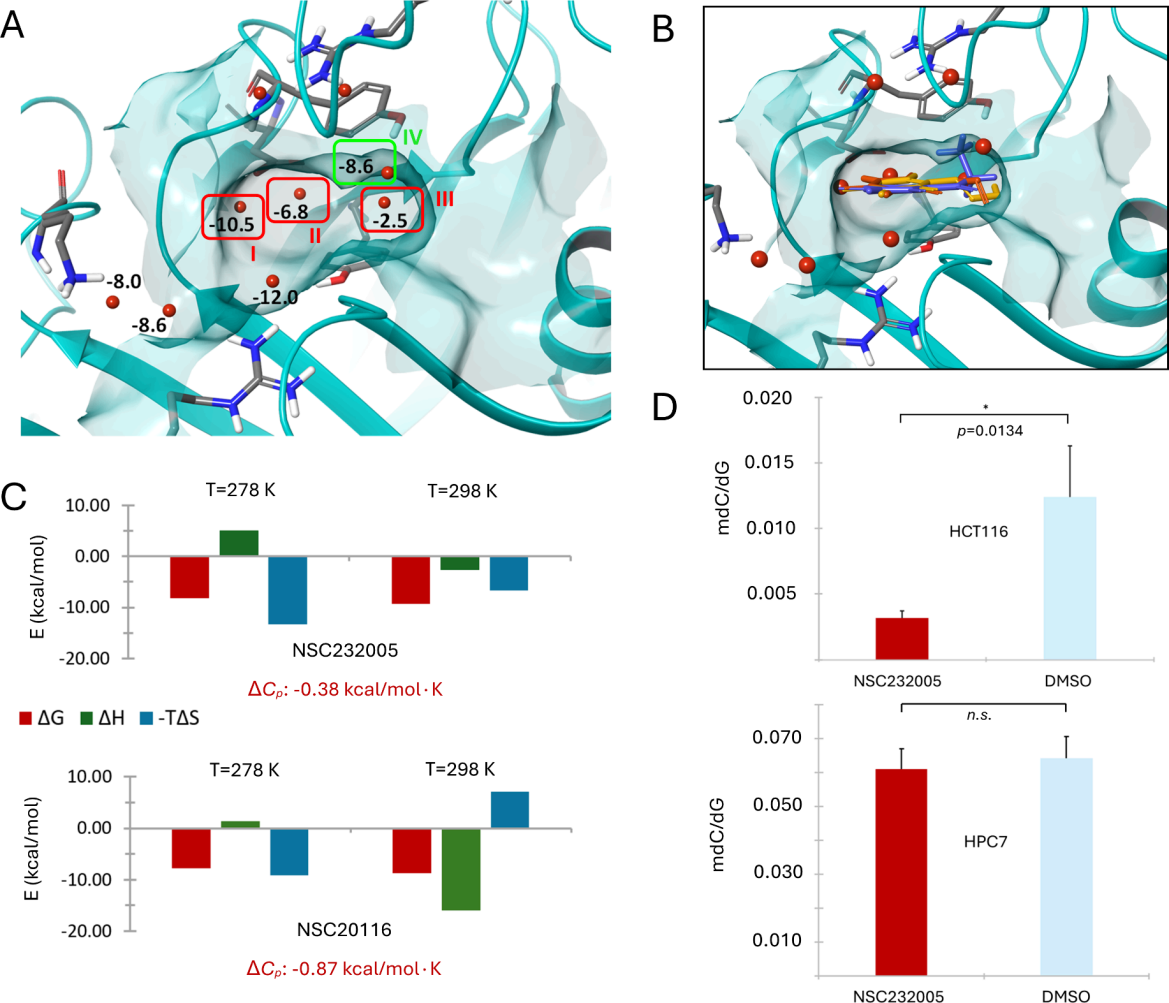
(A) The four stable hydration sites in the 5mC cavity of SRA-UHRF1 according to the SZmap algorithm prediction with their individual Δ*G* scores. (B) The proposed binding mode of NSC232005 (blue), NSC20116-H (yellow) and NSC20116-N (orange) and their overlap with the predicted hydration sites. (C) The thermodynamic profile and individual energy terms of NSC232005 and NSC20116 at two different temperatures based on ITC experiments and the change in heat capacity determined for each ligand–protein system. (D) The effect of NSC232005 at a concentration of 25 μM on the global DNA methylation of colorectal cancer HCT116 cells (*n* = 3, *p* = 0.0134) and healthy hematopoietic HPC7 murine cells (*n* = 4).

### Investigation of Enthalpy–Entropy Compensation Effects

The consensus of simulations indicates as the primary reason for the constructive entropic term determined for NSC232005 the almost equal capacity of its exocyclic substitution low-energy rotamers to bind SRA-UHRF1 efficiently. This was qualitatively shown by the conformational variability of the respective group in the low energy docked poses of NSC232005 (Figure 2A) as well as on the comparably high stability of the same geometries over the metadynamics simulations (Figure 2B), while the particular notion was strengthened by measures derived by MD and mainly on the wide distribution of the corresponding dihedral, which systematically sampled various different *anti* conformations over the three unbiased 1 μs trajectories (Figure S2A, blue radial plots). This lack of conformational preference is accompanied by a similar motif of solvent displacement upon binding for all rotamers (Figure 2A, bottom inset, and Figure 3B), which renders them almost energetically equivalent as to their binding affinity. Specifically, bound ligand dynamics as derived by MD trajectories combined with SZmap mapping indicate at least three stable hydration sites (red waters I–III, Figure 3A) and possibly an additional fourth (green water IV, Figure 3A) competing with the ligand and providing additional evidence for the constructive entropic term determined by ITC. The entropic aspect of NSC232005 binding may also explain the diminished affinity of the highly similar NSC22474 (Δ*T*_m_: 0.73 ± 0.12 °C) which, due to its shorter exocyclic substitution, cannot sustain the displacement of these structural waters from within the 5mC cavity. The destructive entropy of NSC20116 seems, in contrast, to arise from the preferable binding of its hydroxydiazenyl tautomer, which according to *ab initio* calculations and TIMS is more stable in solution, retains a dominant population in the sample and is consistently shown by MD simulations to accommodate stronger and more persistent hydrogen-bond interactions within the SRA-UHRF1 cavity (Figure S2B) compared to the nitroso (mean hydrogen bond count: 4.2 ± 0.5 for NSC20116-H over 3.4 ± 0.5 NSC20116-N). To provide a complementary methodological validation for the binding preference of SRA-UHRF1 toward the hydroxydiazenyl tautomer of NSC20116 over the nitroso, three independent free energy perturbation calculations were performed, with FEP systematically attributing a significantly unfavorable mean change in Δ*G*_binding_ of +1.77 ± 0.3 kcal/mol for the specific mutation (Figure S3). Solvent mapping suggests an additional reason for the unfavorable binding entropy of NSC20116, as the fourth stable hydration site (green water IV, Figure 3A) seems less likely to be displaced by the shorter and more planar exocyclic substitution of either NSC20116 tautomer when compared to NSC232005.

With respect to enthalpic contributions, NSC232005 demonstrates higher fluctuation within the cavity compared to both NSC20116 tautomers as shown in the related RMSD graphs (Figure 2C, bottom inset) as well as on complementary stability measures such as the mean radius of gyration values for the ligand (*R*_g_: 2.9, 2.6, and 2.5, respectively). This attribute in combination with the lower persistence of key hydrogen bonds (Figure S2B) such as the one accommodated by the Asp469 side chain, a part of the thumb DNA-interacting loop of SRA-UHRF1 (Figure S4A), may provide an explanation for the weaker enthalpic term of NSC232005 recorded in ITC experiments. On the other hand, the interaction with Asp469 is augmented by several additional strong hydrogen bonds systematically present in the trajectories of both NSC20116 tautomers and, especially, the hydroxydiazenyl isomer (Figure S2B). The weaker enthalpic interaction of NSC232005 could also be explained by the more intense electropositive potential of the specific cationic ligand (Figure 2D) which is expected to experience considerable Coulombic repulsions against the equivalently electropositive and extended DNA binding interface of the SRA-UHRF1 groove at the entrance of the 5mC recognition cavity (Figure S5) and, moreover, weaker π–π stacking to Tyr466 and Tyr478 as compared to NSC20116. The aspect of optimal fit within the 5mC cavity regarding both confirmed hits should also be emphasized. Similar analogues that carry slightly bulkier substitutions on vectors not pointing toward the solvent, like the aryl sulfonamide NSC22475, fail to retain high affinity (Δ*T*_m_: 0.87 ± 0.12 °C). Diminished affinities of analogues such as NSC22474 and NSC22475 which share high similarity with the hits might additionally be interpreted by variations in their tautomeric equilibria induced by the different substitutions these analogues carry.

### Heat Capacity Measurement

In an effort to rationalize the above-mentioned balance between enthalpic and entropic contributions, the change in heat capacity (Δ*C*_p_) upon ligand binding was determined by repeating the calorimetric analyses at 5 °C (Figure S6). The change in enthalpy revealed unambiguous enthalpy–entropy compensation effects and, as expected, for such systems, afforded negative heat capacity changes^82^ of −0.38 kcal/mol·K (Δ*H*_0_ temperature of 14.5 °C) for the NSC232005/SRA-UHRF1 system and a Δ*C*_p_ of −0.87 kcal/mol (Δ*H*_0_ temperature of 5.9 °C) for the respective NSC20116/SRA-UHRF1 system (Figure 3B). These changes in *C*_p_ in principle indicate more extensive hydrophobic interactions achieved by NSC20116 upon binding to SRA-UHRF1.^83^ The variation of the solvent-accessible surface area (SASA) over the MD trajectories agrees with this notion (Figures S2B and S7). In particular, in the case of NSC232005 solvent-accessible surface removal from bulk solvent is initially low and tends to gradually increase over the simulation time, while for both NSC20116 tautomers the respective SASA is consistently high over the whole trajectories. Nevertheless, from another perspective, the change in *C*_p_ could be counterintuitive on the basis of the additional carbon content in the exocyclic substitution of NSC232005. Interestingly though, similar remarkable cases have been reported in the past where perturbation of an extra structural water between two otherwise similar ligands takes place.^84–86^ Indeed, the unusual and complicated nature of water as a solvent turns heat capacity changes into exceedingly difficult data to interpret solely on the basis of hydrophobicity.^84,87^ In the SRA-UHRF1 system, the SZmap algorithm prediction seems to be in excellent analogy with the case of modified sugars binding to concanavalin-A lectin^85^ where an additional, positionally perturbed (and not displaced) water gives rise to the observed more negative Δ*C*_p_ for the less hydrophobic ligand, although in the con-A case the destructive entropy-enthalpy compensation diminishes any anticipated binding affinity gain for the ligand targeting this additional water. Consequently, in light of the Δ*C*_p_ results the original interpretation of SZmap results needs to be revised, as the fourth predicted hydration site (Figure 3A, green water IV) in the SRA cavity is more likely to be displaced or perturbed by NSC20116 rather than NSC232005. Further contributors to the measured Δ*C*_p_ values were suggested by MD simulations. Different degrees of contact were measured between the ligands and key structural features of SRA-UHRF1 such as the thumb (^463^AGGYEDD^469^) and the NKR finger (^483^GRDLSGNKRTAEQ^495^) DNA-interacting segments (Figure S8), with RMSD values being consistently higher in the NSC232005/SRA-UHRF1 complex (Figure S9), particularly for the thumb loop. Such hydrophobic interactions are expected to have a dominant influence on heat capacity changes and to contribute to the overall binding affinity of each ligand, yet in-depth structural studies are needed to conclusively prove such contributions. The observed Δ*C*_*p*_ could also be argued to agree with the Δ*T*_m_ values determined for the respective ligands, as the less negative Δ*T*_m_ shift determined for NSC232005 could indicate ligand binding to a protein conformational state more closely related to the native folded^88^ and hence amenable to fewer hydrophobic contacts artificially created upon thermal denaturation. The DSF assessment of the two ligands was repeated with a 30 min incubation prior to measurement to check for indications of slow equilibrium steps upon binding, yet no significant Δ*T*_m_ shifts were observed (data not shown).

### In Vitro DNA Demethylation Assessment

Compound NSC232005 was selected for an *in vitro* assessment of its biological activity due to its higher drug-likeness, optimal water solubility, and simpler tautomerism. Its potent binding to the SRA domain suggested the possibility this molecule might inhibit DNA methylation by interfering with the recognition by UHRF1 of hemimethylated DNA strands and subsequent recruitment of DNMT1 leading to inhibition of maintenance methylation. To check this, the global DNA methylation of HCT116 colorectal cancer cells was determined by mass spectrometry using a previously reported protocol.^89^ Colorectal cancer (CRC) is a particularly lethal malignancy where UHRF1 is extensively involved and its inhibition is currently investigated as a promising therapeutic target.^90–92^ With respect to the SRA domain in particular, disruption of its DNA binding capacity has been shown to reactivate tumor-suppressor genes and significantly decrease key CRC oncogenic properties.^48^ Treatment of HCT116 cells with 25 μM NSC232005 for 168 h resulted in a significant DNA methylation decrease of 74.5%, as quantified by the ratio of methylated cytosine (mdC) over unmodified guanine (dG) nucleotides (Figure 3D). This activity could not be reproduced in healthy HPC hematopoietic cells (Figure 3D), providing initial indications of cell type-selective effects, which may imply differences in epigenetic or cell cycle-dependent mechanisms between healthy and malignant cells. Notably, analogous specificity trends toward malignant versus healthy cell lines have been attributed to two structurally different SRA-UHRF1 ligands, compounds AMSA2 and MPB7, with this difference justified on the basis of higher UHRF1 levels in malignant cells.^62^ In terms of cytotoxicity, the effect of NSC232005 has been assessed previously at the NCI yeast anticancer drug screen over different isogenic strains that contain alterations to DNA damage response pathways homologous to human^66,93,94^ where the molecule was inactive, a result suggesting low inherent toxicity and further strengthening its drug-likeness features.

## Conclusions

The promising cell-based activity of our previously reported SRA-UHRF1 ligands prompted a more systematic exploration of uracil derivatives as SRA binders. In this study, a rational stepwise approach was pursued by employing orthogonal biophysical and computational tools as well as a preliminary *in vitro* bioactivity assessment on cells. Possibly due to the nature of those pyrimidine heterocycles, several issues arose at the thermal melt screening stage with respect to safety, toxicity, and potential for rational development. The most promising hits were, however, clearly validated by ITC as potent SRA-UHRF1 ligands, and on the basis of the cell-based DNA demethylation experiments, the most potent of them appears to be an effective UHRF1 inhibitor. In terms of thermodynamics and according to the hypothesis stated here, NSC232005 binding is mainly attributed to a combination of favorable conformational and solvation entropy changes, while NSC20116 binding to stronger enthalpic intermolecular interactions combined with comparable to NSC232005 solvation entropy but with a simultaneous, highly opposing conformational entropy change. In addition to this, the extremely delicate contribution of solvent to the binding affinity of a confirmed ligand is suitably exemplified by the combination of thermodynamic measurements and theoretical simulations in the seeming contradiction between heat capacity changes and compound hydrophobicity.

Although NSC20116 seems more suitable for optimization as reversal of the destructive entropic term is usually thought to be more straightforward,^95^ the presence of a toxic nitroso moiety is a definite drawback for its further development, although this aryl nitrosamine seems to be less prone to undergo Fischer–Hepp rearrangement and subsequent metabolic activation to a carcinogen due to the presence of a carbonyl at the para position of the nitroso substituent on the uracil system.^96^ Another aspect of its suboptimal profile as a lead candidate relates to the considerable likelihood of NSC20116 to be chemically unstable, convert to an alkylating diazonium derivative and act as a covalent binder, or even participate in redox reactions. This notion is reasonable given the presence of the nitroso group, but even if this proves to be the case, well-designed isosteric replacements may mitigate or eliminate this weakness. On the other hand, NSC232005 seems to comply with the primary requisites of a drug lead. Results presented in this study clearly show the bioactivity capacity of this particular pyrimidine core and its potential to sustain medicinal chemistry efforts toward optimizing cell-active DNA methylation modulators targeting UHRF1. A key issue regarding active molecules that originate from biologically privileged molecular scaffolds such as the pyrimidine system is their specificity, which has to be extensively evaluated prior to any major optimization endeavor. Studies at the *in vivo* level are also needed to carefully assess the translational potential of these compounds. In the case that those uracil derivatives prove selective, they may offer original chemical space to sustain the development of highly potent UHRF1-targeting compounds.

In conclusion, the aim of the present study was tandem; first, to characterize in depth the two compounds which were identified via screening and proved to be strong ligands for the SRA domain of UHRF1 and second, to interrogate their binding thermodynamics and present a consistent hypothesis as to the factors determining ligand affinity for the targeted protein. In this direction, the resulting structure–activity relationship notions may serve as a model for guiding the rational design of optimized uracil analogues, which may in turn sustain the development of chemical probes targeting UHRF1 or first-in-class DNA demethylating drugs.

## Materials and Methods

### Protein Expression and Purification

The plasmid encoding the SRA domain of UHRF1 (UBH12, 3CLZ)^25^ was a gift from Cheryl Arrowsmith and Structural Genomics Consortium (Addgene plasmid #25220; http://n2t.net/addgene: 25220; RRID:Addgene_25220). Competent *Escherichia coli* BL21 (DE3) cells were transformed by heat shock and grown at 37 °C to an OD_600_ of approximately 0.5 in Luria–Bertani medium in the presence of 50 μg/mL kanamycin. Protein overexpression was induced by 0.1 mM of isopropyl-β-d-thiogalactopyranoside (IPTG) at 18 °C overnight; cells were harvested by centrifugation and sonicated in lysis buffer, and subsequently the protein was purified in a single step by immobilized metal affinity chromatography (IMAC) using 1 mL of PureCube 100 Ni-NTA agarose of bead size 90–100 μm (Cube biotech 74103) and an elution buffer (HEPES 10 mM pH 7.4, NaCl 300 mM) with 250 mM imidazole. The purified protein was concentrated using Amicon Ultra 2 mL tubes (10 kDa cutoff, Merck Millipore) and its purity was assessed by SDS-PAGE (Figure S10). Quantification of the purified protein was performed at 280 nm by an Implen N50 Nanophotometer. Regardless of the fact SRA-UHRF1 is a DNA-interacting domain, nucleic acid impurities in the purified protein were limited, as determined by the A260/280 nm ratio of 0.62 ± 0.06 (*n* = 5).

### Differential Scanning Fluorimetry

Experiments were performed in a BioRad CFX-Connect real-time PCR machine. Compound screening utilized a continuous heating rate protocol of 0.5 °C/min repeated for 65 cycles after an initial 3 min incubation at 25 °C. The protein was diluted at a concentration of 5 μM in a buffer comprising 10 mM HEPES at pH 7.4 and 150 mM NaCl, while for monitoring protein unfolding the ProteOrange stain reagent (Lumiprobe code 10210) was used in concentrations between 5x and 25x depending on the protein batch. At primary screening, compounds were diluted in the protein buffer from 10 mM stock solutions in 100% DMSO and were evaluated at a concentration of 250 μM. With respect to fluorescence-based compound filtering, compounds NSC22474 and NSC22475 were eliminated as they absorb in the visible region of the spectrum across the yellow-orange frequencies (stock solutions in 100% DMSO were intensely colored), where interference with the orange DSF dye is highly probable. Regarding NSC232002, this derivative was eliminated after it afforded high fluorescence in protein-free control experiments. The dose–response curves of the hits were determined by the same heating protocol at concentrations in a logarithmic scale from 10 to 1000 μM. When described, compound incubation into the protein-stain mix was performed for 30 min in the dark prior to assaying. Analysis and calculation of Δ*T*_m_ values was performed by BioRad Maestro software, Microsoft Excel 365 and the fitting tools of the DSF-World application by the Gestwicki group.^97^

### Molecular Simulations

Docked poses of NSC232005 and NSC20116 in the 5-Me-Cyt pocket of SRA-UHRF1 were obtained by the Glide XP algorithm (Schrodinger Inc.) with Van der Waals radii for protein and ligand nonpolar atoms scaled down to 90% and 80% of their nominal values, respectively. Unrestrained molecular dynamics simulations were performed by using Desmond software (D.E. Shaw Research). The MD systems were prepared by charge neutralizing and solvating the protein–ligand complexes in SPC water and 0.150 M NaCl. Periodic boundary conditions were applied to a triclinic system with a buffer range of 10 Å, and multiple simulations with different random seeds were performed at the NPT ensemble (Nose-Hoover chain thermostat, Martyna-Tobias-Klein barostat) for 1 μs at 298 K. A key issue in the simulations was the ionization state of Asp469 at the 5mC pocket of UHRF1. This residue was predicted to be neutral by the Schrodinger protein preparation algorithm, in spite of chemical intuition and its normal p*K*_a_ value of 3.4. This observation could be partially explained, though, by the almost perfect bidentate interaction formed between the neutral aspartate side chain and the 5mC residue. When, however, an apoprotein structure was considered, the Rosetta-pH method predicted a reasonable mean p*K*_a_ value of 2.5 for the same residue.^98^ Molecular mechanics simulations were performed by using the OPLS-2005 force field. The crystal structure of the SRA-UHRF1 domain bound to a methylated DNA fragment (PDB id: 3CLZ) was used for all simulations. Tautomerism of NSC232005 and NSC20116 was studied by the multistage Jaguar protocol (Schrodinger Inc.) involving the enumeration of all possible tautomeric structures followed by a rough energy sorting at the PM3 semiempirical level, selection of the most stable isomers, and geometry optimization at the DFT level of theory using the B3LYP-D3 functional and the LACVP** basis set and, subsequently, their exact quantum mechanical energy ranking by utilizing the M06–2X functional and a more complete cc-pVTZ­(-f) basis set. For both compounds, atomic partial charges for docking calculations and molecular dynamics simulations were calculated by a thorough minimization at the DTF level with the B3LYP-3D functional and the 6–31G** basis set, followed by a single point energy calculation at the same level of theory with the Poisson–Boltzmann finite element water solvation model and an augmented 6–311G**++ basis set. Solvent mapping was performed by the SZmap algorithm at the “stabilization” calculation mode. All simulations were performed at Linux mint I9 local workstations operating Nvidia 2080 Super and RTX4000 Quadro GPU chipsets.

### Isothermal Titration Calorimetry

A low-volume Nano ITC (TA Instruments) was used for calorimetry experiments. The purified protein was concentrated and buffer exchanged using Amicon Ultra 2 mL tubes (Merck Millipore). The ITC protein buffer consisted of 10 mM HEPES at pH 7.4 and 150 mM NaCl adjusted to 0.1% DMSO for matching compound samples. All samples were degassed prior to titration under a high vacuum. Isotherms were obtained by reverse titrations where the cell was filled with approximately 300 μL of ligand at 10 μM diluted in the same buffer used for concentrating the protein and the syringe was filled with 50 μL of protein at 120 μM matched with the addition of 0.1% DMSO. Titrations were performed at 5 and 25 °C. For titrations, 25 injections of 2.5 μL each (first injection: 0.95 μL) with an interval of 180 s between injections were utilized at a stirring rate of 300 rpm. Blank experiments were performed by titrating the ligand sample into protein buffer, and dilution heats were checked for linearity and low heat signal. Analysis was performed by NanoAnalyze software using an independent binding model. Baseline correction in the case of linear blank experiments was performed by fitting the last 5 points of each ligand to protein experiment to a constant and subtracting this value to obtain corrected heats.

### Compound Preparation - Assessment

All compounds were provided free of charge from the NCI/DTP repository (htpps://dtp.cancer.gov) with the exception of 5-methyl-2’-deoxycytidine-5’-triphosphate which was purchased as a sodium salt (Jena Biosciences NU-1125S). The NCI/DTP compounds were dissolved in DMSO and 5-methyl-deoxycytidine triphosphate was dissolved in distilled water at a concentration of 10 mM. Solutions were stored at −20 °C. The active compound assessment in terms of structural identity and purity was performed by high-resolution mass spectrometry (HRMS). The base peak chromatogram of NSC232005 displayed a single peak eluting at 0.87 min, indicating chromatographic purity under the applied conditions (Figure S11). The corresponding high-resolution mass spectrum acquired in negative ionization mode exhibited a base peak at *m*/*z* 168.0412 which was consistent with the molecular formula C_6_H_7_N_3_O_3_. The calculated ring double bond equivalent was 4.5, and the mass error was −1.76 ppm, supporting the proposed structure and confirming the compound identity. Further structural confirmation was provided by the HRMS/MS spectrum which displayed a predominant fragment ion at *m*/*z* 125.0355 (base peak, 100% intensity), corresponding to the formula C_5_H_6_N_2_O_2_ and consistent with the expected fragmentation pattern. With respect to NSC20116, for the extracted ion chromatogram corresponding to the *m*/*z* of 155.0211 a single chromatographic peak eluting at a *t*_R_ of 1.3 min was observed. This *m*/*z* was assigned to the neutral molecular formula C_4_H_4_N_4_O_3_ with a mass accuracy of −0.1 mDa/–0.5 ppm and an isotopic fit of 15.2 mSigma using the Smart Formula Manual (Data Analysis, Bruker Daltonics, Germany). The two dominant peaks observed in the mobilogram of the *m*/*z* 155.0211 correspond to mobility values of 0.813 and 1.147 V·s/cm^2^, respectively. Analysis of NSC20116 was performed using ultra high-performance liquid chromatography (UHPLC) (Elute LC series, Bruker Daltonics, Germany) coupled to a hybrid trapped ion mobility-quadrupole time-of-flight system (TIMS-QTOF) powered by PASEF (timsTOF Pro, Bruker Daltonics, Germany). An Acquity UPLC BEH C18 (Thermo Fisher Scientific, Germany) equipped with a Van guard Acquity UPLC BEH C18 (Waters, Ireland) was selected for the chromatographic analysis at 30 °C. The analysis was conducted in negative electrospray ionization mode and the mobile phases consisted of H_2_O:MeOH (99:1 v/v) with 5 mM ammonium acetate and 5 mM ammonium acetate in MeOH. For the UPLC-HRMS analysis, a Waters Acquity H-Class UPLC system (Waters, USA) coupled to a Velos Pro-Orbitrap Elite hybrid mass spectrometer (Thermo Fisher Scientific, USA) was employed. Chromatographic separation was carried out on a Supelco Ascentis Express C18 reversed-phase column at 40 °C. Mass spectrometric detection of NSC232005 was performed using a heated electrospray ionization (HESI) source operating in both positive and negative ion modes. The capillary and heater temperatures were 350 °C. High-resolution mass spectra were acquired using Xcalibur 4.6 and processed with Freestyle 1.8 software (Thermo Fisher Scientific, USA).

### Cell Culture

HCT116 cells were cultured in McCoy’s 5A medium (Gibco) supplemented with 10% fetal calf serum (Life Technologies) and penicillin–streptomycin antibiotics at 140 and 400 μg/mL, respectively. They were routinely tested for mycoplasma contamination. For drug treatment, 5 × 10^5^ HCT116 cells were seeded in a 6-well plate and treated with at 25 μM NSC232005 or DMSO for 7 days. Media and inhibitor were replenished every 48 h. After a 96 h incubation period, cell viability was assessed using the Trypan Blue exclusion assay. Following viability assessment, cells were washed with 1 x PBS and cell pellets were collected. These pellets were subsequently processed for DNA extraction according to the manufacturer’s protocol provided in the QIAGEN DNA extraction kit AllPrepDNA/RNA Mini (Cat. No. 80204). Cell culture media: IMDM-base media (Life Technologies Ltd., 12440061), 150 mM monothioglycerol (Merck Life Science UK Limited, M6145–25 ML), 1× penicillin-streptomycin (Gibco, 15140122), 1× l-glutamine (Gibco, 25030081), 5% fetal bovine serum (PANBiotech, P30–3306), and 10% SCF conditioned DMEM media (CHOK3 cells produced Stem Cell Factor). HCT116 cells were cultured in McCoy’s 5A (Gibco) supplemented with 10% fetal calf serum (FCS, IGC technical services) and 2% penicillin streptomycin (Pen/Strep, IGC technical services). Cells were usually grown in T75 flasks and passaged every 2–3 days, when the cells reached 70–90% confluency. Cells were passaged by aspirating old media followed by a wash with phospate-buffered saline (PBS, IGC technical services). Cells were trypsinised by adding TrypLE Express (Gibco) and incubated for 5 min at 37 °C. Trypsin was then inactivated by the addition of ten volumes of culture media containing FCS. The mixture was then pipetted until single-cell suspension was achieved. A fraction of the cell suspension was placed in a T75 flask containing the fresh media. Cells were passaged at a 1:10–1:2 seeding ratio depending on the initial confluency. Collection of cell pellets was performed when the cells were 70–80% confluent. Cells were trypsinised by adding TrypLE Express (Gibco) and incubated for 5 min at 37 °C. Trypsin was then inactivated by the addition of ten volumes of culture media containing FCS. Cells were then centrifuges at 1000 rpm for 5 min. Pellets were then washed with ice cold PBS and centrifuged again at 1000 rpm for 5 min. PBS was then aspirated, and the dry pellets were quickly placed on dry ice sprayed with ethanol.

### In Vitro DNA Methylation Assay

As a first step, nucleic acid hydrolysis was performed. The yield of genomic DNA was quantified by a Qubit Fluorometer 3 (Thermo Scientific) at 150 ng for 5-methylcytosine and hydrolysis was undertaken by 2.5 μL of 10× Degradase Reaction buffer (Zymo Research, E2020) and 0.5 μL of Degradase Plus enzyme (Zymo research, E2020) per sample (5 IU) with the volumes adjusted with HPLC-grade water (VWR Chemicals, 83645.320P). The reaction was incubated at 37 °C for 3 h. Samples were added to 0.2 μM filtration plate (Agilent, 203940–100) mounted on a V-shaped 96-well (Agilent, 5043–9313) MS plate. The samples were centrifuged at 3220 g for 35 min and 4 °C. Flow-through was measured and the volume in each well was adjusted up to 40 μL with water or buffer A. Standards of 13 μL were loaded onto the plates and sealed the V-shaped, 96-well plate with sealing mat (Agilent, 5043–9317). Nucleic acid quantification was done on an Agilent 6495 triple quadrupole LC/MS system and a Zorbax C18 column (1.8 μm, 2.1 mm 150 mm; Agilent Technologies, 859700–902). Data was acquired in dMRM mode with injection volume 5 μL injected in replicates. Mobile phase in isocratic gradient was Buffer A (ammonium acetate of 0.77 gr, acetic acid 99% of 90 μL for a pH of 6, HPLC grade water up to 1 L) and Buffer C (100% methanol) with proportions at 5 min 100% A, 0% C and by 17 min 0% A, 100% C.

## Supplementary Material



## Data Availability

The crystal structure of SRA-UHRF1 (pdb code: 3CLZ) was downloaded from PDB (www.rcsb.org). Docking was performed using Glide (version 91117, MMshare version 54117), the metadynamics analysis was performed by the binding pose metadynamics protocol of Maestro (version 12.8.117, MMshare version 5.4.117), molecular dynamics and free energy perturbation were performed by academic Desmond (version 2022–4, multisim version 4.0.0, MMshare version 6.0), solvent mapping was done using SZMAP (version 1.6.6.1) and *ab initio* simulations using Jaguar (version 11.2). Docked poses, docking grids, input and output files of MD simulations, metadynamics and FEP calculations, SZMAP results and ligand structures can be freely found at Github (https://github.com/vmyriant/Data_and_software).

## Notes

Structures of screened compounds, MD measurements (dihedral angles, distances, RMSD values, hydrogen bond counts), FEP results, *ab initio* analysis of intramolecular hydrogen-bonding of NSC232005 and NSC20116, ITC raw and fitted results at 5 °C, structural features and electrostatic potential of SRA-UHRF1, SDS-PAGE of protein samples, and active compound LC-HRMS and HR-MS/MS analyses (PDF)
