# Supplementary material for: Discovery and Characterization of Uracil Derivatives Targeting the Set-and-Ring Domain of UHRF1
Source: J Chem Inf Model. 2025 Aug 19;65(17):9274–86. doi: 10.1021/acs.jcim.5c01345 (PMC12421671; doi:10.1021/acs.jcim.5c01345)
Supplement: Supplementary file 1 [file ci5c01345_si_001.pdf]

Supplementary Information

Title:

Discovery and characterization of uracil derivatives targeting the Set-and-Ring domain of UHRF1.

Authors: Ifigeneia Akrani[1], Danai Driva[1], Efstratios Tsakalidis[1], Nandini Mozumdar[2], Danai Mavridi[1], Anthi Panara[3], Kalypso Epiphaniou[1], Maria Chalkiadaki[1], Andreanna Frances Wright[4], Maria Halabalaki[1], Angeliki Tsoka[5], Grigoris Zoidis[1], Constantinos Vorgias[5], Duncan Sproul[4], Evangelos Gikas[3], Skirmantas Kriaucionis[2], Emmanuel Mikros[1], Vassilios Myrianthopoulos[1*].

Affiliations:

[1] Division of Pharmaceutical Chemistry, Department of Pharmacy, National and Kapodistrian University of Athens, Panepistimiopolis Zografou 15771, Athens, Greece.

[2] Ludwig Institute for Cancer Research, Nuffield Department of Medicine, Old Road Campus Research Building, OX3 7DQ, Oxford, United Kingdom.

[3] Laboratory of Analytical Chemistry, Department of Chemistry, National and Kapodistrian University of Athens, Panepistimiopolis Zografou 15771, Athens, Greece.

[4] Medical Research Council Human Genetics Unit, Institute of Genetics and Cancer, University of Edinburgh, EH4 2XU, Edinburgh, United Kingdom.

[5] Department of Biology, National and Kapodistrian University of Athens, Panepistimiopolis Zografou 15771, Athens, Greece.

†: These authors contributed equally

Corresponding author: Vassilios Myrianthopoulos, vmyriant@pharm.uoa.gr, Division of Pharmaceutical Chemistry, Department of Pharmacy, National and Kapodistrian University of Athens, Panepistimiopolis Zografou 15771, Athens, Greece, +30 210 7274353.

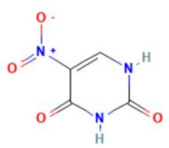

NSC9790

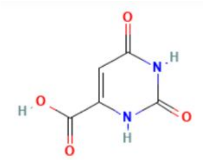

NSC9791

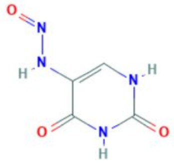

NSC20116

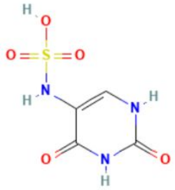

NSC20117

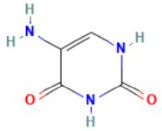

NSC22474

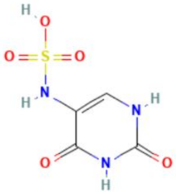

NSC22475

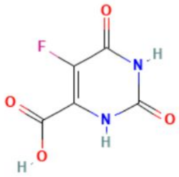

NSC31712

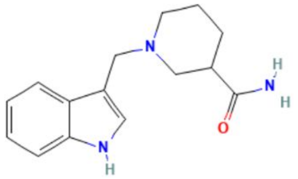

NSC32474

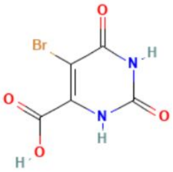

NSC34493

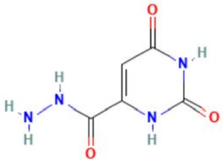

NSC34715

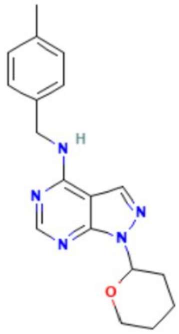

NSC50265

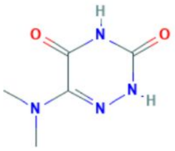

NSC107682

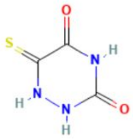

NSC107684

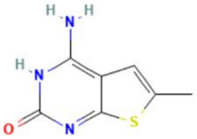

NSC180529

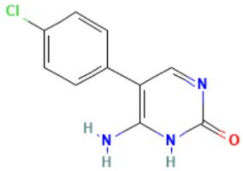

NSC211371

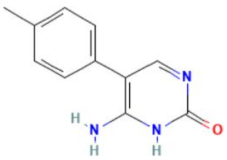

NSC211565

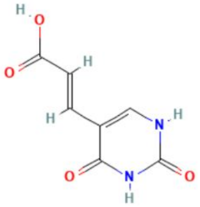

NSC232002

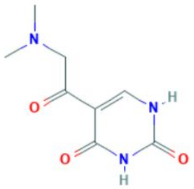

NSC232004

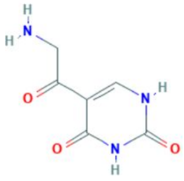

NSC232005

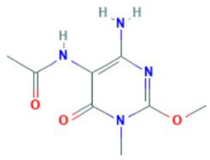

NSC622776

Table S1. The structures of the NCI/DTP compounds assessed in the thermal melt screening stage as potential SRA-UHRF1 ligands with their corresponding NSC identification numbers; compounds NSC20117 and NSC22475 are identical.

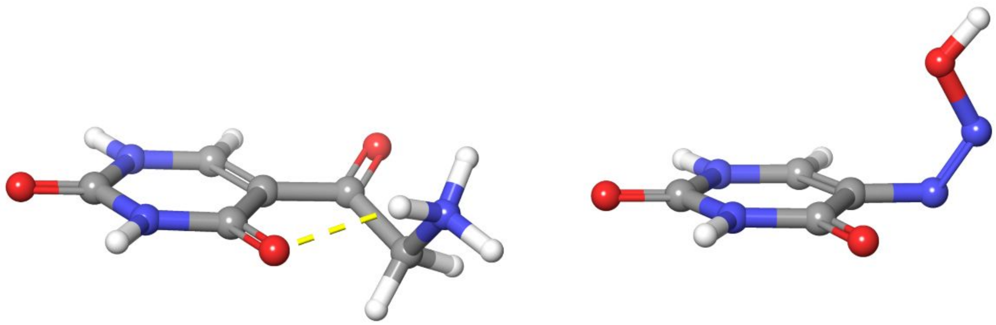

Figure S1. The conformations of compound NSC232005 (left) and the diazenyl tautomer of NSC20116 (right) resulting after a minimization at the molecular mechanics level of theory (OPLS 2005) and a subsequent *ab initio* energy calculation at the DFT level (B3LYP-D3 functional, Poisson-Boltzmann finite element solvation model and a 6-311**++ basis set) of conformers derived by manual dihedral rotations aiming to enforce intramolecular hydrogen bonding. Concerning NSC232005, the specific conformer was less stable by +1.67 kcal/mol from its non-bonded counterpart (shown in the main text and used for all simulations). Concerning NSC20116-H, forced application of a *cis* double bond isomer could not afford any structures with valid intramolecular hydrogen bonding but instead resulted to the extended conformation demonstrated hereby which possessed a ΔE of +8.99 kcal/mol compared to the *trans* isomer used for all calculations in the main manuscript text.

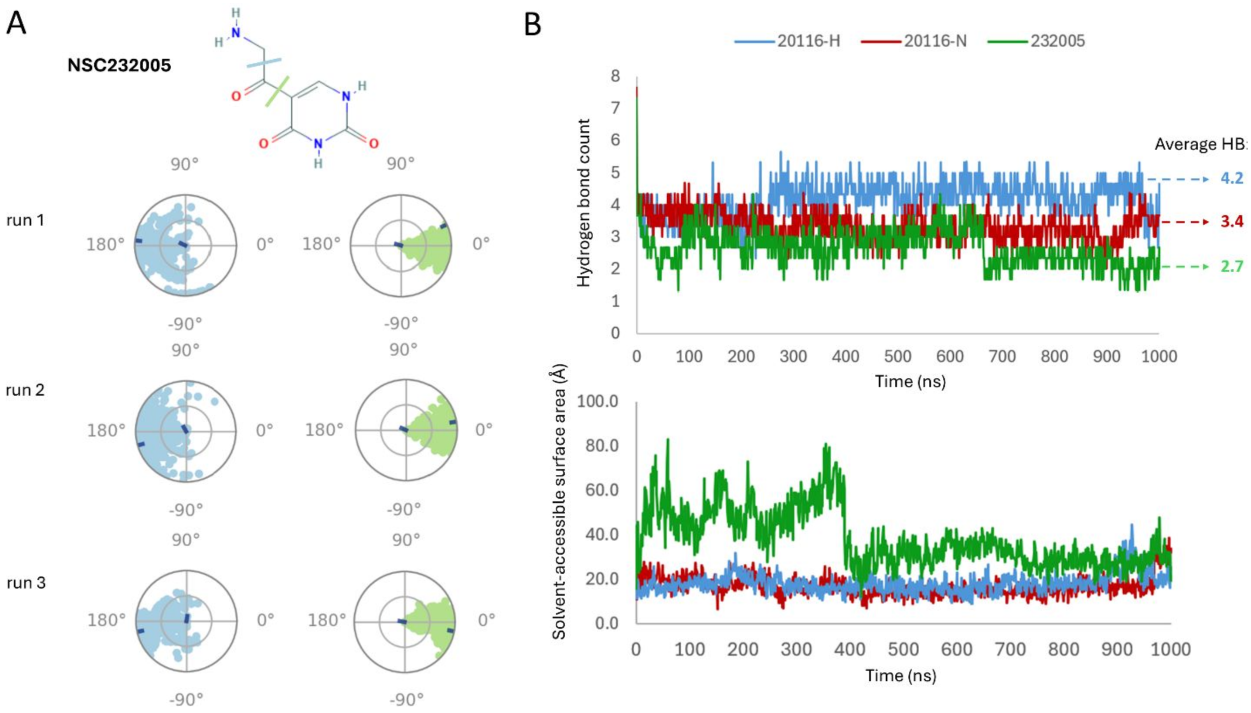

Figure S2. A) Radial plots demonstrating the distribution of the two dihedral angles of the NSC232005 exocyclic substitution (shown by light blue and green lines on the two-dimensional structure) as monitored throughout the three replica 1 µs unbiased MD simulations; B) Graphs of the mean values of intermolecular hydrogen bond count (top) and ligand solvent-accessible surface areas (bottom) monitored throughout the three replica 1 µs unbiased MD simulations for each ligand-protein complex (color-coded in legend).

	$\Delta\Delta G$ (kcal/mol)	SD
trajectory 1	+1.78	± 0.10
trajectory 2	+2.09	± 0.10
trajectory 3	+1.43	± 0.11
mean	+1.77	± 0.33

Figure S3. The free energy perturbation results in terms of the change in $\Delta G_{binding}$ and its standard deviation for the three replica mutations of the hydroxydiazenyl tautomer of NSC20116 to its nitroso tautomer in the 5mC binding pocket of SRA-UHRF1.

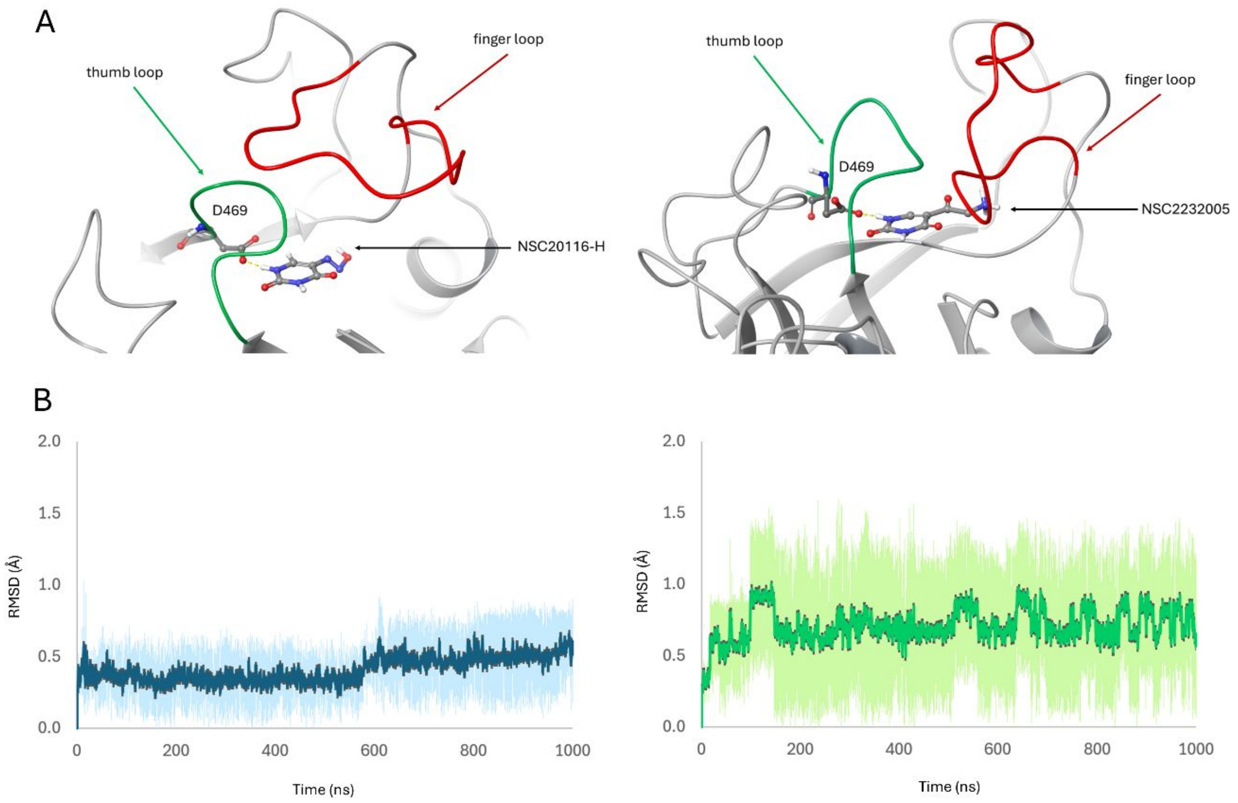

Figure S4. A) The location of Asp469 within the 7-residue long NKR thumb DNA-interacting segment (green ribbon) with respect to the position of the 13-residue long finger DNA interacting segment (red ribbon) and the 5mC binding pocket in complex with NSC20116-H (left) and 232005 (right). A key hydrogen bond between the carboxylate Asp469 sidechain and each respective ligand is shown as a yellow dashed line; B) The mean values (dark colors) and standard deviations (light intervals) of the Asp469 RMSD from starting coordinates in the NSC20116-UHRF1 (left) and 232005-UHRF1 (right) complexes over the three replica 1 μs MD simulations.

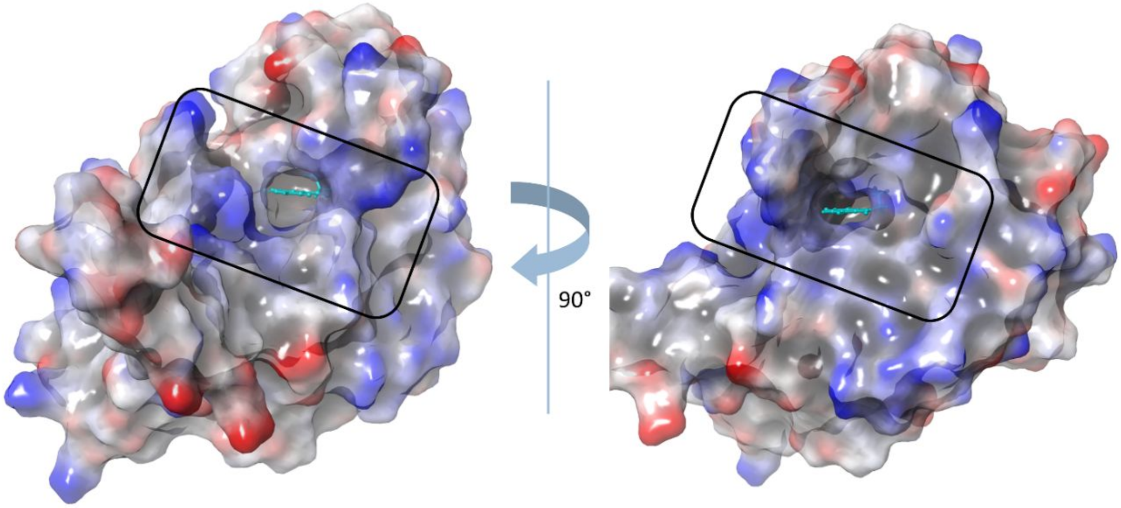

Figure S5. The electrostatic potential of the SRA-UHRF1 mapped on the 0.001 a.u. electron density isosurface (blue surface: positive charge, red surface: negative charge) around the 5mC recognition cavity which is indicated by a tilted rectangle. The intensely positive potential around the 5mC cavity is well anticipated given the strongly negative charges of the native DNA ligand.

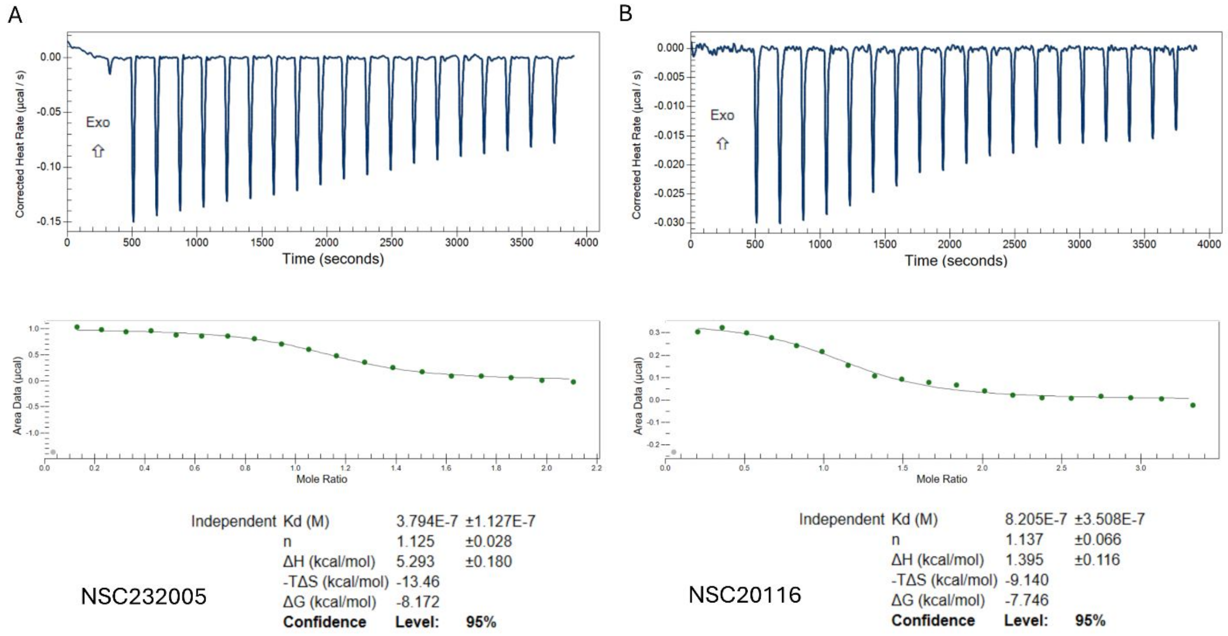

Figure S6. The ITC thermograms, fitted models and derived thermodynamic data for the interaction of compounds NSC232005 (A) and NSC20116 (B) with the SRA-UHRF1 as determined at a temperature of 5 °C.

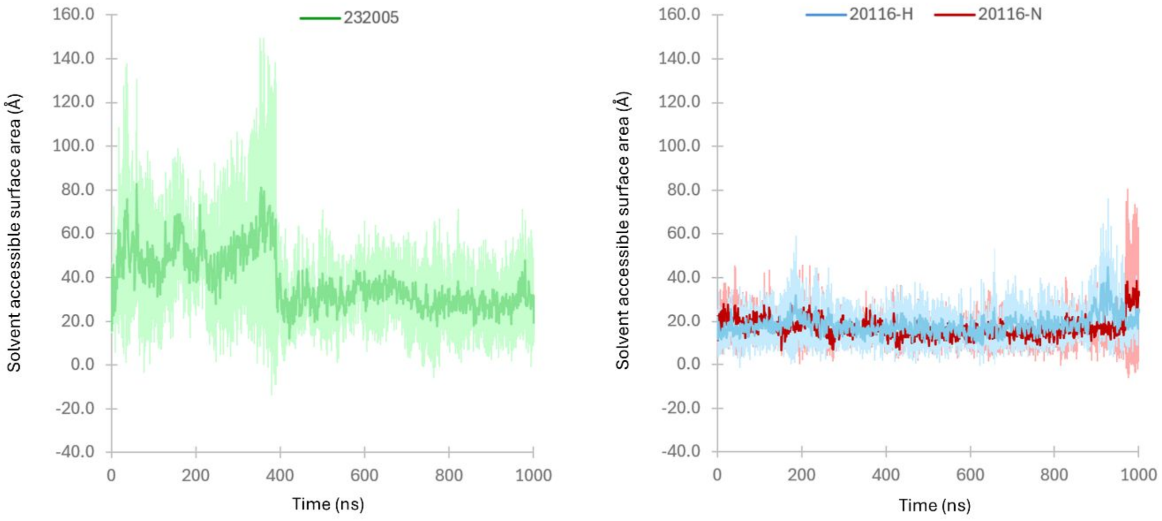

Figure S7. The mean values (dark colors) and standard deviations (light intervals) of the ligand solvent-accessible surface areas (SASA) over the three replica 1 µs MD simulations for each respective ligand-protein complex. The SASA of NSC232005 tends to decrease over the simulation time thus indicating a gradually increasing ligand burying whereas the preclusion of NSC20116 solvent-accessible surfaces from the bulk solvent is achieved almost completely from the beginning of the trajectories.

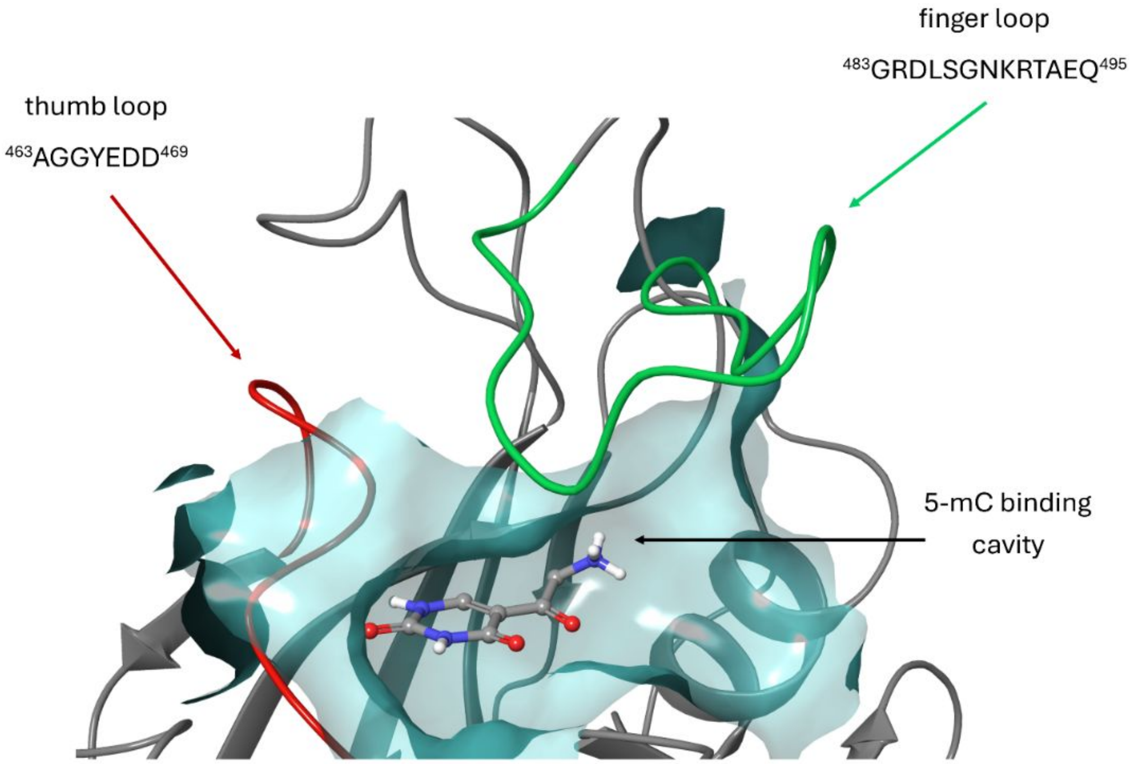

Figure S8. The position of the two DNA-interacting SRA-UHRF1 segments with respect to the position of the 5mC binding pocket which is shown as a cyan-colored molecular surface in complex with compound NSC232005.

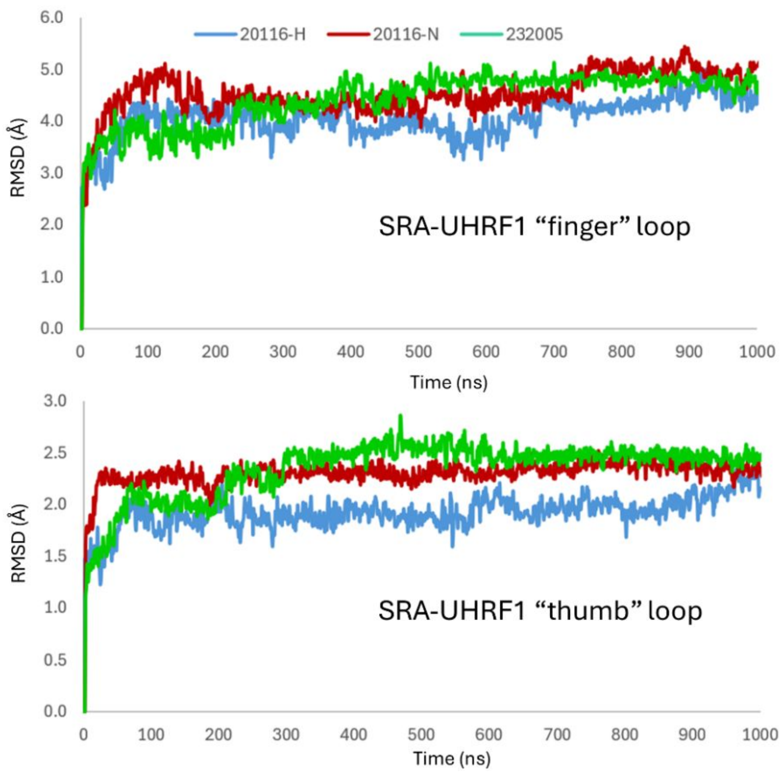

Figure S9. The mean values of the root-mean-square deviation of the finger (upper graph) and thumb (lower graph) DNA-interacting domains of SRA-UHRF1 from starting coordinates over the three replica 1 μs unbiased MD simulations in the respective ligand-protein complexes. In both cases the fluctuation of the DNA-interacting loops, and particularly the thumb, is higher in the NSC232005-UHRF1 complexes suggesting the existence of weaker hydrophobic interactions between the protein and the ligand.

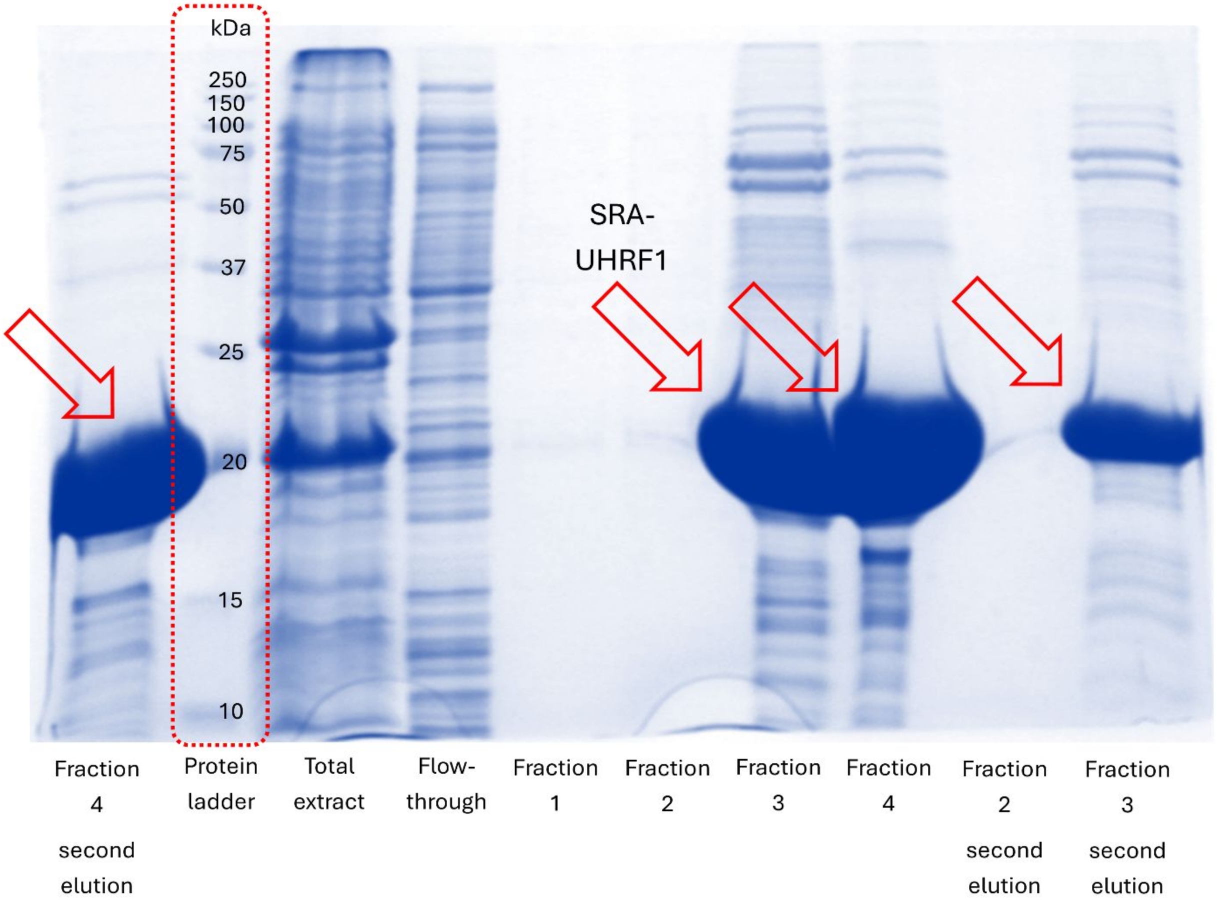

Figure S10. The SDS-PAGE gel of the SRA-UHRF1 domain purification process. The corresponding protein marker is depicted in column 2. The SRA construct encodes a polypeptide with a MW of 22743.4 Da. The domain was expressed in high yield.

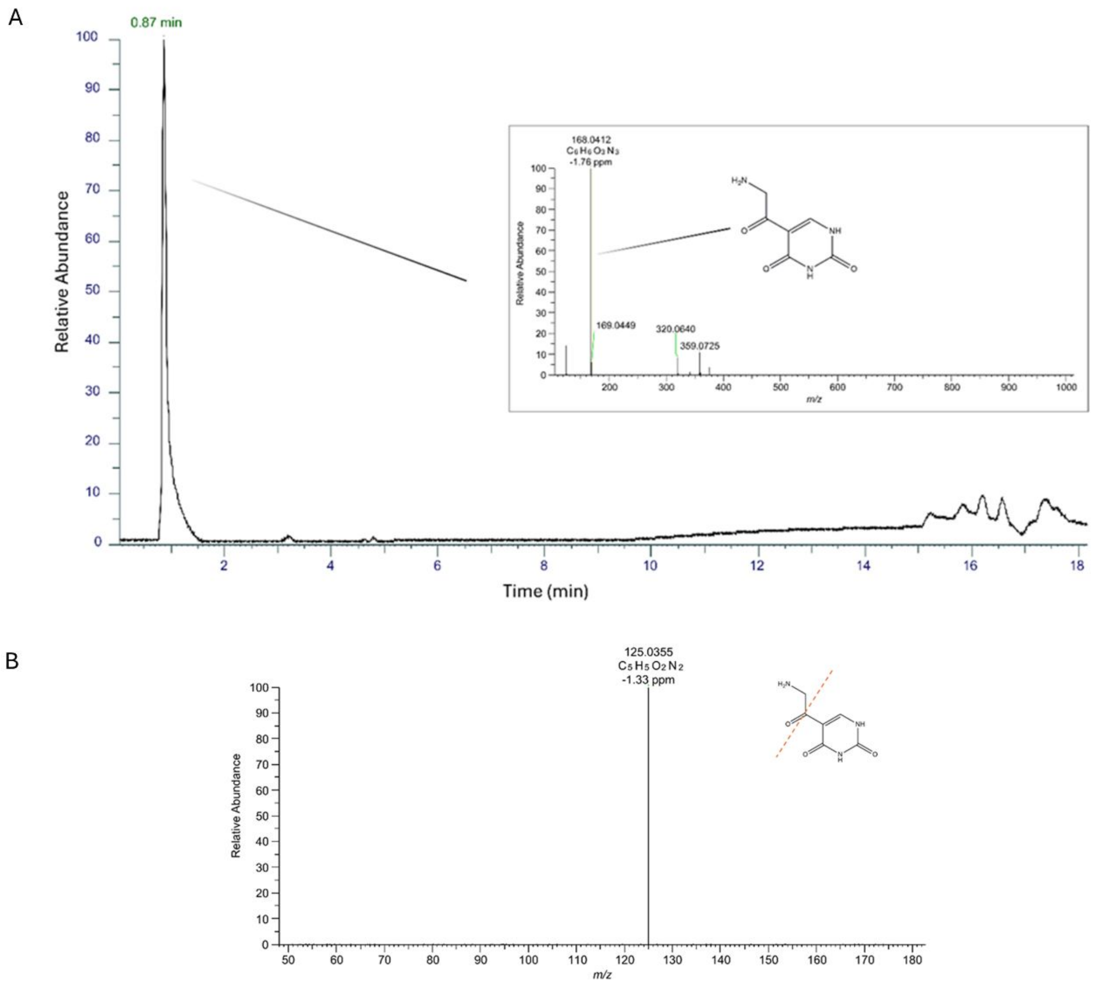

Figure S11. (A) The base peak chromatogram of compound NSC232005 obtained by LC-HRMS displays a single peak eluting at 0.87 min, indicating chromatographic purity under the applied conditions. The corresponding high-resolution mass spectrum acquired in negative ionization mode exhibits a base peak at *m/z* 168.0412 which is consistent with the molecular formula $C_6H_7N_3O_3$. The calculated ring double bond equivalent is 4.5 and the mass error is -1.76 ppm, supporting the proposed structure and confirming the compound identity. (B) Further structural confirmation is provided by the HRMS/MS spectrum, which displayed a predominant fragment ion at *m/z* 125.0355 (base peak, 100% intensity), corresponding to the formula $C_5H_6N_2O_2$, consistent with the expected fragmentation pattern.
